# Spent Coffee Grounds in the Production of Lightweight Clay Ceramic Aggregates in View of Urban and Agricultural Sustainable Development

**DOI:** 10.3390/ma12213581

**Published:** 2019-10-31

**Authors:** Fernanda Andreola, Alessandro Borghi, Simone Pedrazzi, Giulio Allesina, Paolo Tartarini, Isabella Lancellotti, Luisa Barbieri

**Affiliations:** Dipartimento di Ingegneria “Enzo Ferrari”, Università degli Studi di Modena e Reggio Emilia Via Vivarelli, 10/1–41125 Modena, Italyalle.borghi@hotmail.it (A.B.); simone.pedrazzi@unimore.it (S.P.); giulio.allesina@unimore.it (G.A.); paolo.tartarini@unimore.it (P.T.); isabella.lancellotti@unimore.it (I.L.)

**Keywords:** spent coffee grounds, lightweight expanded clay aggregates, fertilizer glass, urban and agricultural sustainable development

## Abstract

This paper presents an innovative application for spent coffee grounds (SCGs) used as filler for the formulation of lightweight clay ceramic aggregates (LWA). LWA can be used for urban and agricultural purposes as a sustainable solution. Spent coffee grounds were tested as a pore forming agent partially acting as a replacement for red clay in material formulation before firing. Substitutions of 10, 15, and 20 wt.% of red clay were tested. The properties of lightweight aggregates with 15 wt.% of SCGs were improved using a specifically tailored fertilizer glass due to its low pH and conductivity within the soil tolerance range. Packaging glassy sand, cattle-bone flour ash, and potassium carbonate were mixed and melted in order to obtain this glass, which when added to the clayey batch functionalized the aggregates by phosphorus and potassium nutrients. The results (in particular, porosity and bulk density) show that the lightweight aggregates obtained have interesting properties for possible uses both in urban (e.g., green roofs as a drainage layer) and agricultural purposes. Moreover, pH and conductivity are in line with the Italian Standard regarding soil amendment (D.lgs. 75/2010). In addition, several leaching tests were performed in a solution containing 2 vol.% citric acid (C_6_H_8_O_7_) to evaluate the release capacity not only of nutrients (P and K) but also to check the presence and release of heavy metals, such as lead (Pb), that may come from the glassy precursor. The results obtained showed that nutrients are efficiently released in 21 days (P = 87.73% and K = 25.74% of released percentage) and Pb release is under the standard threshold of 30 ppm.

## 1. Introduction

One of the most widely consumed hot beverages worldwide is brewed coffee. In the crop season 2017–2018, the total production by all exporting countries of green coffee beans reached 9513 million tons [1]. According to the European Coffee Report 2017–2018 [2], Europe is the second-largest worldwide green coffee importer, with about 3425 million tons imported in 2017. 

The tradition of coffee roasting in Italy is well established and appreciated abroad. According to ISTAT data [3], Italy exports roasted coffee of about 4 million equivalent green bags (60 kg/bag), an increase of 4.9% compared to 2016. The roasted coffee is directly delivered and sold to the clients, mostly bars, where it becomes espresso, and then spent coffee grounds (SCGs) are generated as post-consumer products. SCGs are commonly treated as organic waste and sent to composting plants or in the unsorted garbage that it is fired in incinerator power plants [4]. However, SCGs have multipls interesting properties for a wide range of applications—anaerobic co-digestion with different waste feedstocks for biogas production [5,6], composting processes [7], nutrient for fungi growing [8], nutrient for ruminant feed [9], fertilizer for agricultural soil [10], ethanolic extraction [11], adsorption of pollutants for water treatments [12], geopolymers [13], etc. A SCGs-based biorefinery for the production of biofuels, biopolymers, antioxidants, and biocomposites is studied in the literature [14]. On the other hand, as reported elsewhere [15], an energy re-use of SCGs, through oil extraction and making pellets, is proposed. The oil derived from the SCG and the solid residue after extraction can be used to produce biodiesel and electrical energy using the system proposed by Allesina et al. [16]. In addition, the solid residue post oil extraction can be pelletized in order to use it as a more flexible fuel in a downdraft stratified gasifier [17,18,19]. A circular economy model based on SCG pelletization and combustion applied to a roasting company was presented by Allesina et al. [20]. However, other and new opportunities arise for this post-consumer product; thinking about the ceramic sector and analyzing the characteristics of SGCs, the high organic matter presence could be exploited for lightening an aggregate material, while the high calorific power could contribute to lower the firing temperature and the fuel consumption of the latter. Since clay ceramic materials are a class of products with a robust market and a significant environmental impact from their manufacture to their disposal, the incorporation of residues as component elements can lead to a new generation of materials that are well placed within the concept of a circular economy (in particular as regards the recycling aspect). Among ceramic products, lightweight aggregates (LWAs) are becoming highly interesting. They belong to the aggregate family, the second most consumed material by man after water [21], amounting to 2282 million tons in Europe in 2016 [22], given that its sector is the main supplier of raw materials for the construction of infrastructures and buildings, as well as for industry and environmental protection, which confers it a clearly strategic character [21]. The physical and chemical characteristics of aggregates make them suitable for several applications—lightweight concrete, green roofs, substrates in horticulture, hydroponic substrate, gardening in general, or others, such as geotechnical applications, pavements, or filtering media, depending on the structure and properties obtained. In particular, lightweight aggregate is the generic name for a group of aggregates having a lower density than normal aggregates like natural sand, gravel, and crushed stones. For instance, industrial expanded clay consists of a granular lightweight aggregate obtained by subjecting special natural clays to a thermal expansion and vitrification (clinkerisation) process at temperatures higher than 1200 °C [23]. The granules have a lightweight internal cellular structure with good insulating properties. This is enclosed within a compact, strong external shell that provides an excellent weight/strength ratio, making the product suitable for a wide range of geotechnical, infrastructure, and construction applications. This product is also durable and fireproof material, it is frost resistant, thermally insulating, and soundproof [24]. Among the performances to be tested, porosity is essential to give lightness and a draining effect to the material, as well as the ability to release nutrients over time. These are key aspects for the expanding market of LWAs to urban and industrial green management, as well as water and air treatment applications; landscaping (green roofs and roof gardens, urban landscaping, playing fields); horticulture, floriculture and gardening (cultivation in pots and planters, mulching, plant reproduction); hydroculture (hydroponics, intensive soilless cultivation, indoor and outdoor cultivation, aquaponics); and water and air treatment (constructed wetlands and phyto-purification, sludge tanks cover, filtration). For instance, the construction of new roof gardens and green roofs (both extensive and intensive) is continuously increasing because of the many advantages they bring both to new buildings and refurbishments [25]. The key to green roof and roof gardens is the lightweight material used as a drainage layer. This material is able to retain water while having some fertilization properties. The term fertilizer includes substances that, due to their content in nutritive elements or their chemical, physical, or biological characteristics, contribute to the improvement of the fertility of the agricultural soil or contributes to the nutriment of cultivated plants. Fertilizers provide the chemical elements of fertility necessary for the plants to carry out their vegetative and productive cycle [26]. Soil improvers modify the chemical, physical, biological, and mechanical properties and characteristics of a land, thus improving its habitability for cultivated plant species. 

Another interesting aspect related to lightweight aggregates is the possibility to use very different recovery raw materials from spent glauconite [27,28,29], etc.

The paper includes preliminary studies of tailoring, realization and characterization of new lightweight expanded ceramic aggregates (LWAs), based on spent coffee grounds as a pouring agent, functionalized by a fertilizer glass tailored by the authors and containing cattle-bone flour ash, packaging glassy sand, and potassium carbonate. Evaluations of some chemical-physical parameters such as pH, conductivity, porosity, density, static and dynamic water absorption were performed on the obtained spherical samples. Results of the analyses confirm that the samples are suitable for the above-mentioned purposes. 

## 2. Materials and Methods

### 2.1. SCGs Collection, Drying, and Characterization

About 5 kg of spent coffee grounds (SCGs) were collected from a coffee bar located near the engineering campus of the University of Modena. The coffee bar owner participated in the collection putting SCGs in dedicated bins. The collected SCGs were very humid (65% of water content in weight). To avoid mold and fungus propagation, the SCGs were immediately dried spreading them out on a plastic canvas in a ventilated room at a temperature of about 20 °C. After 48 h, the water content of the SCGs dropped to 7% in weight, enough to reduce the risks of mold and fungus formation. Moisture content of SCGs was evaluated by weighing a sample of coffee before and after the drying process in a Memmert Universal Oven UF30 for 24 h at 103 °C according to the standard ASTM D4442-15(2015). Elemental composition of a dry SCGs sample (Table 1) was assessed using a Thermo Fisher Scientific Elemental CHNS-O analyzer (model FLASH 2000). The Higher Heating Value (HHV) of the dry sample is calculated using Equation (1) [30].

HHV_dry_ = 0.3491C+1.1783H+0.1005S-0.1034O -0.0151N-0.0211ASH (1)

where C, H, S, O, N, and ASH (wt.%])are the weight fraction of carbon, hydrogen, sulphur, oxygen, nitrogen, and ash of the dry SCGs sample. ASH content was assessed by calcination in oven at 550 °C for 4 h according to the standard ASTM E1755-01(2015).

### 2.2. LWAs Preparation

LWAs were prepared by substituting from 10 to 20 wt.% of a local ferruginous red clay with SCGs previously described (named SCG10, SCG15, and SCG20). Red clay contains higher silicon quantities, typical for this material, as well as alumina (Al_2_O_3_), corresponding to higher refractoriness, and iron oxide (Fe_2_O_3_ = 7.89%) corresponding to red color. From mineralogical point of view, quartz (SiO_2_), kaolinitic, muscovitic and chlorite clays, calcium and magnesium carbonate (Dolomite ((Ca, Mg) CO_3_)) and Hematite (Fe_2_O_3_) as chromophore oxide were identified. Many details related to used clay are already reported in Farias et al. [31] Moreover, a functionalization of the aggregates with 15 wt.% of SCGs (named SCG15FG10, SCG15FG30, SCG15FG0) has been performed by adding from 10 up to 50 wt.% of a fertilizer glass (FG) tailored by the authors in order to obtain LWAs with a (K_2_O+P_2_O_5_) wt.% content of 12 wt.% (glass matrix fertilizer) according to Italian Regulation D.Lgs. 75/2010 art 1 [26]. The fertilizer glass was made using glassy sand as matrix and adding cattle bone flour ash and potassium carbonate as sources of P and K, respectively. Glassy sand is an Italian secondary raw material (commercial product) obtained by the second treatment of that fraction of packaging glass cullet not destined for glassworks and so codified as waste (19 12 05 European Waste Code), in turn deriving from the primary treatment of packaging glass from urban collection. The chemical composition is SiO_2_ = 71.70, Al_2_O_3_ = 2.25, Na_2_O = 12.50, K_2_O = 1.00, CaO = 9.50, MgO = 2.00, BaO = 0.04, SrO = 0.37, Fe_2_O_3_ = 0.43, and TiO_2_ = 0.07. In order to obtain a suitable paste, the clay and SCGs were ground and sieved at 1 mm, and the fertilized glass was ground in an Agatha ball mill and sieved below 100 µm.

Spherical samples of 1.5–2.0 g of weight and 1–1.5 cm of diameter were handmade from the mix of raw materials moistened with appropriate water content (20–30%) in order to obtain the adequate plasticity required for shaping. Then, the samples were dried in stove at 105 ± 5 °C in order to remove the free water from the body, so avoiding cracking during firing, which takes place inside a static furnace (Lenton AWF13/12) at a temperature of 1000 °C according to Farias et al. [31]. Once the set temperature has been reached, the aggregates inside refractory crucibles are placed into the oven and fired for one hour. This process subjects the aggregates to a thermal shock similar to that what they would suffer in industrial processes generally occurring at higher temperatures (from 1200 to 1400 °C). After firing, the samples are extracted and left to cool at room temperature for 12h. The samples were codified as reported: red clay + spent coffee ground (SCG followed by a number indicating the percentage of SCGs introduced) and red clay + spent coffee grounds + fertilizer glass (SCGFG followed by a number indicating the percentage of fertilizer glass added to a base composition opportunely chosen of 85% of clay and 15% of SCGs).

### 2.3. LWA_S_ Characterization

The sintered clay materials were subjected to different physical-chemical tests in order to determine their possible use in agriculture or building sector. The following tests were done: X-ray fluorescence (XRF ARL-ADVANT‘XP + THERMO equipment, software, Uniquant Thermo Fisher Scientific Inc. Waltham, Massachusetts, US) analysis of raw materials (red clay, SCGs and FG); water absorption capacity (W.A.%) by immersion of samples in boiling water (100 °C) for 6 h, according to UNE-EN 772-7 (1999); static water absorption in cool distilled water after 24 h follow UNI EN 772-21 (2011); bulk density (BD) by Envelope density analyzer Geo Pyc 1360 equipment (Micromeritics, 4356 Communications Drive Norcross, GA30093-2901, USA); absolute or real density RD by Gas Helium-pycnometer Accupyc TM II 1340 (Micromeritics 4356 Communications Drive Norcross, GA30093-2901, USA); total porosity percentage (TP), by means of the Equation 2; pH value, according to UNI-EN 13037 (2012) standard, using a pH meter Oakton CON6 (OAKTON Instruments P.O. Vernon Hills, Il, USA); electrical specific conductivity (S.C), according to UNI-EN 13038 (2012) standard, using an Oakton conductimeter CON6/TDS6 (OAKTON Instruments P.O. Vernon Hills, Il, USA)).

(2) TP = [(RD−BD)/RD] × 100

Besides, LWAs sample with FG were evaluated by X-Ray Diffraction (XRD) analysis (X ’PERT PRO, PAN Analytical, Malvern Panalytical Ltd., Malvern, UK) with Ni-filtered Cu Kα radiation operating at 40 mA and 40 kV. For the qualitative analysis, data were recorded in the 5–70° 2q range (step size 0.02° and 0.5 s counting time for each step). The qualitative analysis was conducted by XPert High Score Plus software (Malvern Panalytical Ltd. Malvern, UK). For the analysis of LWAs surface microstructure, a scanning electron microscope ESEM Quanta-200 was used coupled with a system for microanalysis X-EDS INCA-350 (Oxford Instruments, Tubney Wood, Oxfordshire, UK). For ESEM analysis, samples were pasted on an individual aluminum sample-holder with silver paste and covered by sputter coating with an Au/Pd source (K550 Emitech Ltd. South Stour Avenue, Ashford, UK).

### 2.4. LWA_S_ Release Test

Only for LWAs with fertilizer capability, specific tests of release were performed to evaluate the release capacity of nutrients (P and K) as well as of elements that for their quantity and type could be harmful for the environment. The tests were performed according to both Italian and European regulations, respectively [26,32] in different conditions (distilled water and citric acid solution 2 vol.%) at two time frames (30 min to ensure the immediate nutrients release and 21 days to verify controlled release). The aggregates were tested in whole size, to simulate the conditions occurring in the soil. The liquid solutions derived were analyzed by inductively coupled plasma mass spectrometry (ICP-MS Agilent 7500a, 5301 Stevens Creek Blvd, Santa Clara, CA95051, USA).

## 3. Results and Discussion

### 3.1. LWAs Characterization and Ceramic Properties 

Raw materials chemical analysis (XRF) are reported in Table 2. The composition of the red clay shows a high content of silica (SiO_2_) and alumina (Al_2_O_3_), characteristic components of clayed material. The presence of iron (Fe_2_O_3_), calcium (CaO), and magnesium (MgO) oxides identifies this clay as a ferruginous-calcareous clay responsible for the development of the red color of the products after firing. The tailored fertilized glass shows silicon, calcium, potassium, and phosphorus oxides as its main components. On the basis of the European [32] and Italian [26] Guidelines, the minimum requirements in P and K content for glassy matrix fertilizer are K_2_O + P_2_O_5_ ~12% and K_2_O ≥ 5%; P_2_O_5_ ≥ 5%. The high content in potassium and phosphorous oxides K_2_O + P_2_O_5_ ~30% allows us to functionalize the LWAs with an adequate nutrients content to become fertilizer. Mineral fertilizers are some of the most important products for the agricultural industry. They provide the chemical elements necessary for plants to carry out their vegetative and productive cycle. Fertilizers play an important role in regulating both the pH and the fertility of the soil. SCGs show a high Lost of Ignition (L.O.I.) due to the large amounts of organic compounds contained (fatty acids, amino acids, polyphenols and polysaccarides). Inorganic elements, where potassium is the main, followed by silicon, phosphorus and magnesium, correspond to only 2 wt.% contained in the ash [33]. The realization of glass-containing aggregates started from the study of the aggregates with different percentages of SCGs (10, 15, and 20 wt.%) used as a pore forming agent. These aggregates are compared to another performant clayey LWA previously studied by the authors, containing 15 wt.% of brewery sludge (BS15) and chosen as reference [31]. The experimental data are listed in Table 3.

**Figure 1:**
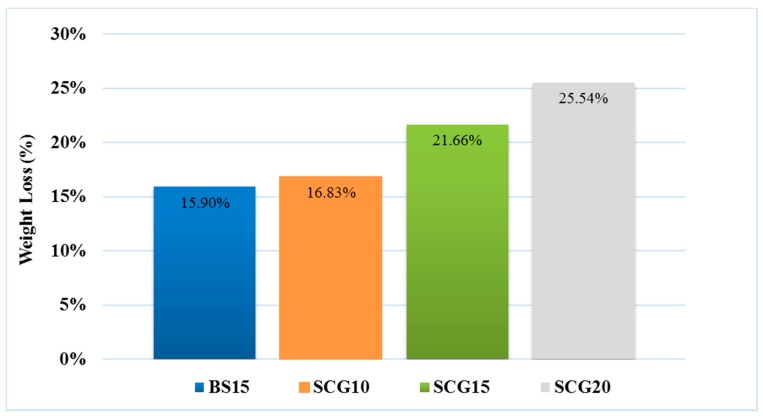
Weight loss (W.L.) for LWAs containing spent coffee grounds (SCGs) at different percentages compared to LWAs containing brewery sludge (BS) at 15 wt.%.

LWAs obtained using SCGs show interesting properties to be used for example in green roofs as a drainage layer. In particular, weight loss during the firing process (W.L.%) and W.A.% confirm lightness and porosity higher than sample containing BS, as shown in Figure 1. For agronomic use pH and specific conductivity are the most important parameters to check. All these mixes are in line with the soil guidelines (6.5 < pH < 7.5 and 0.2 < S.C. < 2 mS/cm). The presence of coffee slightly decreases pH and conductivity—only for 20 wt.%, an increase is observed. The drainage effect is particularly good for the sample containing 20 wt.% of SCGs, to which correspond the highest porosity (over 57%) and the lowest density (around 2.63 and 1.12 g/cm^3^ for real and bulk density, respectively). The sample containing 15 wt.% of SCGs, SCG15, was chosen to be functionalized with fertilizer glass due to the low pH and conductivity values within the soil tolerance range. As appears evident by the data listed in Table 3, the presence of glass into the composition improves the sintering of LWAs causing an increase in bulk density and a slight decrease in W.A.% and porosity with respect to the reference SCG15, but the drainage effect is already significant. Moreover, for agronomic use, pH and specific conductivity of all these mixes are in line with the above-mentioned soil guidelines, and no significant variation is observed. For both the samples series, the RD, BD, and porosity values obtained are comparable with other paper using different pore forming agent [29].

**Figure 2:**
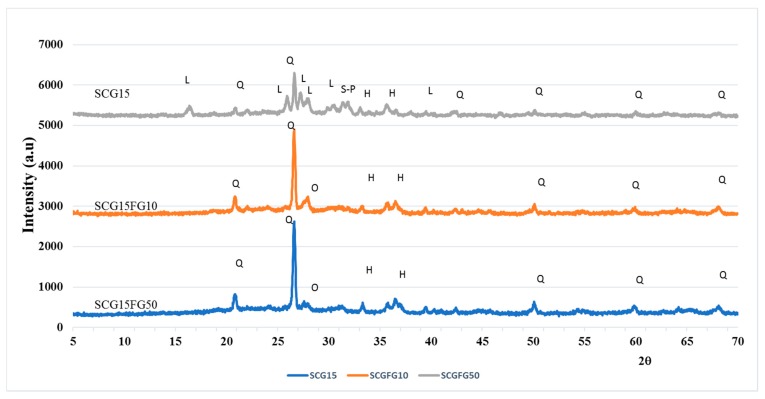
X-ray diffraction (XRD) patterns of LWA_S_SCG15, SCGFG10, and SCGFG50. FG = fertilizer glass, Q = quartz SiO_2_, H = hematite, Fe_2_O_3_ S-P = sodium calcium phosphate silicate, Na_2_Ca_4_(PO_4_)2SiO_2_ L = leucite KAlSi_2_O_6_; hematite, O = orthoclase KAlSi_3_O_8_.

With the aim to study the mineralogical composition of the LWAs, X-ray diffraction analysis was performed. In Figure 2, the patterns of the aggregates SCG15, SCG15FG10, and SCG15FG50 are reported. The main crystalline phases identified for SCG15 and SCG15FG10 samples are quartz SiO_2_ and orthoclase (KAlSi_3_O_8_), and hematite (Fe_2_O_3_) is also observed. This suggests that the addition of a small percentage of glass fertilizer into the mixture does not change the mineralogy of the compounds. Therefore, it can be concluded that the sample prepared with 10 wt.% of glass has the same crystalline phases as the ceramic matrix. Instead, the SCG15FG50 sample, containing a high quantity of glass, presents different mineralogical species such as quartz (Q); sodium calcium phosphate silicate (S-P) Na_2_Ca_4_(PO_4_)2SiO_2_ derived from the fertilizer glass; potassium aluminosilicate, leucite (L) KAlSi_2_O_6_; and hematite (H).

**Figure 3:**
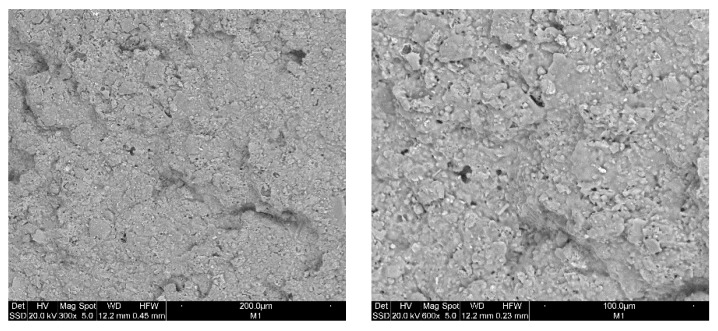
Scanning electron microscopy (SEM) micrographs of SCG15: (left) 300×; (right) 600×.

**Figure 4:**
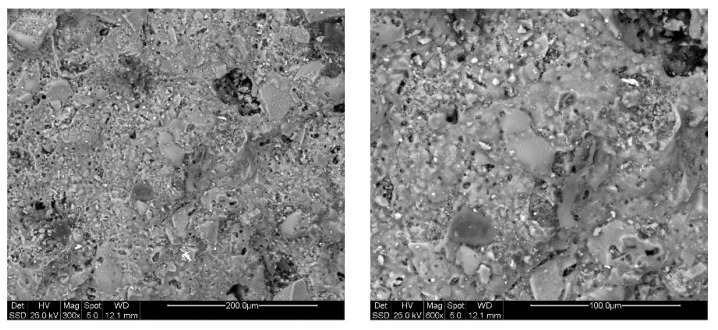
SEM micrographs of SCGFG50: (left) 300×; (right) 600×.

Using the scanning electron microscope (ESEM), it is possible to carry out investigations relating to the morphology and microstructure of materials (Figure 3 and Figure 4) to analyze shape and size of grains porosity, defects and inclusions present. The EDS probe has been used to identify the elements present inside the material and to make a semi-quantitative chemical analysis. The sample containing the pore forming agent (spent coffee grounds) in Figure 3 shows a surface with porosity both spherical and irregular. Spherical pores have dimensions of a few microns, while irregular pores present higher dimensions. The found microstructure is quite similar to those found by W. Franus et al. [27] for lightweight aggregate containing spent glauconite, while M. Franus et al. [29] find a more porous matrix for aggregate containing used motor oil. The surface of SCGFG50 sample (Figure 4), i.e., that containing the maximum percentage of fertilizer glass, shows a non-homogeneous structure with glassy grains and clayey areas well distinguishable from the microstructural point of view and also from semiquantitative analysis. Indeed, in the vitrified area P content is around 2.1 wt.% (obtained by the average of four areas), being glass formulated with phosphorous, while the clay area is poorer in P, which is around 0.7%. The glassy grains have dimension of about 50 µm surrounded by a liquid phase (smooth areas) corresponding to partially melted glassy grains. The presence of glassy grains after the firing process and the not completed dissolutions of them are important topics because they guarantee the slow release of P and K. The amount of these elements is significantly lower with respect the amount in the starting glass, confirming the melting process occurring in the glassy grains. Despite the high content of glass fertilizer in this formulation, it still has porosity, as confirmed by a low apparent density (1.41 g/cm^3^) only slightly higher with respect the value (1.24 g/cm^3^) of same sample without the addition of fertilizer glass (SCG15). This is an important feature for designing lightweight materials with the property of nutrient release. Further, porosity calculation shows a value of 43.6% with respect to SCG15 sample, which shows a value of 55.5%, confirming the presence of significant porosity (notwithstanding the presence of glass). Having analyzed the surface of the samples, the porosity is an open porosity, confirming the high water absorption of the sample, which allows for the release of nutrients.

### 3.2 Release Test of LWAs

The data reported are relative to test performed in citric acid 2 vol.% using whole granulates in two time frames to evaluate the released capacity of nutrients (P, K), other important elements for the growth of plants, and Pb (coming from the glassy precursor). The sample containing 50 wt.% of FG (SCG15FG50) was chosen for the tests because it is the only composition with the minimum quantity of nutrients required by the Italian and European guidelines for fertilizers. From the results shown in Table 4, it is possible to observe that, in 21 days, there is the almost total release of phosphorus (87%) and a quarter (25%) of potassium, while after 30 min, only a very low amount is released. Regarding the lead release, after 21 days, the 44% of the total content is reached (corresponding to 248 ppb = 0.248 mg/L); considering that the limit in fertilizers for Pb is 30 ppm according to Italian regulation D.Lgs 75/2010, this is a low value. On the basis of the results obtained, it is possible to observe that the LWAs functionalized with FG play a role of controlled release fertilizers (CRFs) [34]. In fact, after 21 days, the amount of K and P solubized is greater with respect to 30 min.

## 4. Conclusions

This study reports a novel method for the waste management of spent coffee grounds by valorization them in added value materials, thus avoiding the landfill option. The results indicate the potential for manufacturing high-quality lightweight clay aggregates for draining purposes, especially using spent coffee grounds at 15 wt.% mixed with clay. Regarding mixes containing spent coffee grounds with a fertilizer glass appropriately engineered by the authors, good results of pH, conductivity, and release of nutrients were found, confirming a positive effect of the material on the soil. The release tests would indicate that these functionalized LWAs play a role in the controlled release of fertilizer.

## Figures and Tables

**Table 1:** Elemental composition and Higher Heating Value (HHV)_dry_ of dry spent coffee grounds (SCGs).

C [wt.%]	H [wt.%]	N [wt.%]	S [wt.%]	O [wt.%]	ASH [wt.%]	HHV_dry_ [MJ/kg]
48.67	6.54	2.27	0	40.03	2.43	20.48

**Table 2:** Chemical analysis (XRF) of the materials used.

Oxide [wt.%]	Red Clay	SCGs	FG
SiO_2_	52.77	0.37	31.85
Al_2_O_3_	17.95	0.03	3.85
Fe_2_O_3_	7.89	0.02	0.23
MgO	3.88	0.17	1.44
K_2_O	2.85	0.58	12.10
CaO	2.57	0.19	26.50
TiO_2_	0.78	0.01	0.06
Na_2_O	0.66	0.14	6.19
MnO	0.19	0.00	0.00
P_2_O_5_	0.00	0.32	17.50
L.O.I (1050 °C)	9.90	98.05	0.00
TOTAL	99.44	99.88	99.72

**Table 3:** Characterization for lightweight clay ceramic aggregates (LWAs) containing SCG at different percentages compared to LWA containing brewery sludge (BS) and LWAs containing 15 wt.% of SCGs and different percentages of fertilizer glass (FG).

MIX	W.A. [wt.%] (Boiling Water)	W.A. [wt.%] (Static Water)	pH	Specific Conductivity S.C. [mS/cm]	Real Density [g/cm^3^]	Bulk Density [g/cm^3^]	Total Porosity [%]
BS15	26.45	18.70	7	0.453	2.764	1.410	48.98
SCG10	25.23	15.48	6.7	0.193	2.569	1.416	44.89
SCG15	37.77	17.14	6.8	0.171	2.781	1.237	55.51
SCG20	46.78	30.16	7.2	0.265	2.629	1.124	57.24
SCG15FG10	20.90	15.09	6.7	0.28	2.73	1.34	50.88
SCG15FG30	18.53	14.29	6.9	0.19	2.57	1.40	45.35
SCG15FG50	15.88	12.04	6.8	0.17	2.49	1.41	43.63

**Table 4:** Release test results (elements % with respect to the total amount contained in the material) in citric acid (C_6_H_8_O_7_) at 30 min and 21 days for SCG15FG50 composition.

Elements [wt.% of Release]	30 min	21 days
Si	0.17	7.59
Al	0.20	36.04
Na	0.00	29.54
K	0.07	25.74
Ca	0.64	78.54
Mg	0.84	37.39
P	0.91	87.73
Fe	0.05	11.77
Pb	0.35	44.41
